# Improving disease surveillance data analysis, interpretation, and use at the district level in Tanzania

**DOI:** 10.1080/16549716.2022.2090100

**Published:** 2022-08-02

**Authors:** Irene R. Mremi, Calvin Sindato, Coleman Kishamawe, Susan F. Rumisha, Sharadhuli I. Kimera, Leonard E.G. Mboera

**Affiliations:** a SACIDS Foundation for One Health Sokoine University of Agriculture, Morogoro, Tanzania; b National Institute for Medical Research, Headquarters, Dar es Salaam, Tanzania; c Department of Veterinary Medicine and Public Health, Sokoine University of Agriculture, Morogoro, Tanzania; d Tabora Research Centre, National Institute for Medical Research, Tabora, Tanzania; e Mwanza Research Centre, National Institute for Medical Research, Mwanza, Tanzania; f Malaria Atlas Project, Geospatial Health and Development, Telethon Kids Institute, Perth Children’s Hospital, Western, Nedlands, Western Australia, Australia

**Keywords:** Disease surveillance, data analysis and use, early warning, district, Tanzania

## Abstract

An effective disease surveillance system is critical for early detection and response to disease epidemics. This study aimed to assess the capacity to manage and utilize disease surveillance data and implement an intervention to improve data analysis and use at the district level in Tanzania. Mapping, in-depth interview and desk review were employed for data collection in Ilala and Kinondoni districts in Tanzania. Interviews were conducted with members of the council health management teams (CHMT) to assess attitudes, motivation and practices related to surveillance data analysis and use. Based on identified gaps, an intervention package was developed on basic data analysis, interpretation and use. The effectiveness of the intervention package was assessed using pre-and post-intervention tests. Individual interviews involved 21 CHMT members (females = 10; males = 11) with an overall median age of 44.5 years (IQR = 37, 53). Over half of the participants regarded their data analytical capacities and skills as excellent. Analytical capacity was higher in Kinondoni (61%) than Ilala (52%). Agreement on the availability of the opportunities to enhance capacity and skills was reported by 68% and 91% of the participants from Ilala and Kinondoni, respectively. Reported challenges in disease surveillance included data incompleteness and difficulties in storage and accessibility. Training related to enhancement of data management was reported to be infrequently done. In terms of data interpretation and use, despite reporting of incidence of viral haemorrhagic fevers for five years, no actions were taken to either investigate or mitigate, indicating poor use of surveillance data in monitoring disease occurrence. The overall percentage increase on surveillance knowledge between pre-and post-training was 37.6% for Ilala and 20.4% for Kinondoni indicating a positive impact on of the training. Most of CHMT members had limited skills and practices on data analysis, interpretation and use. The training in data analysis and interpretation significantly improved skills of the participants.

## Background

Over the years, the integrated disease surveillance and response (IDSR) in Sub-Saharan Africa has relied heavily on the data generated from the routine health management information system (HMIS) implemented at the facility and district levels of the health systems [1–8]. The HMIS operates through a web-based open-source District Health Information Software-2 (DHIS2) to capture real-time data on diseases and health service utilisation [9]. The DHIS2 has proved to be a useful tool in supporting the availability of data for strategic planning, priority setting and making effective decisions and public health responses [10,11]. One of the objectives of the IDSR that has not been sufficiently addressed is an improved data analysis and use of information for decision-making [7,12–15]. This is largely dependent on skills, capability and practices related to data management including analysis, visualization, interpretation, and use.

Like elsewhere in Sub-Saharan Africa, studies in Tanzania have found that routine analysis of surveillance data at the health facility and district levels is not optimal, attributed mainly to lack of adequate analytical capacity and skills among the healthcare providers and managers [7,16–18]. All these observations indicate that the analysis and response components of disease surveillance system in Tanzania is weak. Thus, the existing system and surveillance information are not effectively used to monitor disease trends and initiate responses at the primary healthcare levels.

The Tanzanian health management system is decentralised, i.e. most the decisions on disease surveillance are made at the district level. It is critical to have skilled workforce at this level that can manipulate, analyse, and interpret surveillance data generated within their level to support decision making. However, the perception on data quality and usefulness, lack of skills, complex information systems, and the volume, velocity, variety, veracity and value hinder these practices [19,20]. It is important therefore, to establish appropriate and sustainable strategies to monitor such processes ensuring that those involved are adequately capacitated to access the data, conduct basic analysis and perform correct interpretation, as well as generate evidence to support their actions. Among the approaches could be devising a continuous mechanism to identify skill-gaps, provision of repetitive hands-on data management training programmes coupled with building skills on visualization, identifying important patterns, interpretation, and continuous monitoring of trainees. This study aimed to assess the capacity to manage and utilize disease surveillance data and implement a training intervention designed to improve data analysis and use at the district level in Tanzania.

## Methods

### Study site and setting

This study was conducted in Ilala and Kinondoni districts in Dar es Salaam City, Tanzania. Dar es Salaam is the largest commercial city in Tanzania with an area of 1,339 km^2^. According to the 2012 national census, the city had a population of 4,364,541, with an annual growth rate of 5.6% [21]. At the time of this study, Ilala district had a total of 189 healthcare facilities of which 153 (80.9%) were dispensaries, 19 (10.1%) health centres and 17 (9.0%) hospitals. Kinondoni had 106 (74.7%) dispensaries, 10 (7.0%) health centres and 26 (18.3) hospitals.

In Tanzania, each district has a council health management team (CHMT) responsible for overseeing implementation of health activities under the supervision of the regional health management team (RHMT). The routine health management information system (HMIS) is the primary source of data for IDSR and monitors large number of other indicators [8]. Structure-wise, within the CHMT, there are focal persons for each key programme/department in the district, including the HMIS and IDSR. The two focal persons are responsible for analysing and interpreting the data generated from HMIS and IDSR, presenting to the district management in a manner that facilitates utilization in decision-making process.

### Study implementation and data collection processes

**Figure 1.**
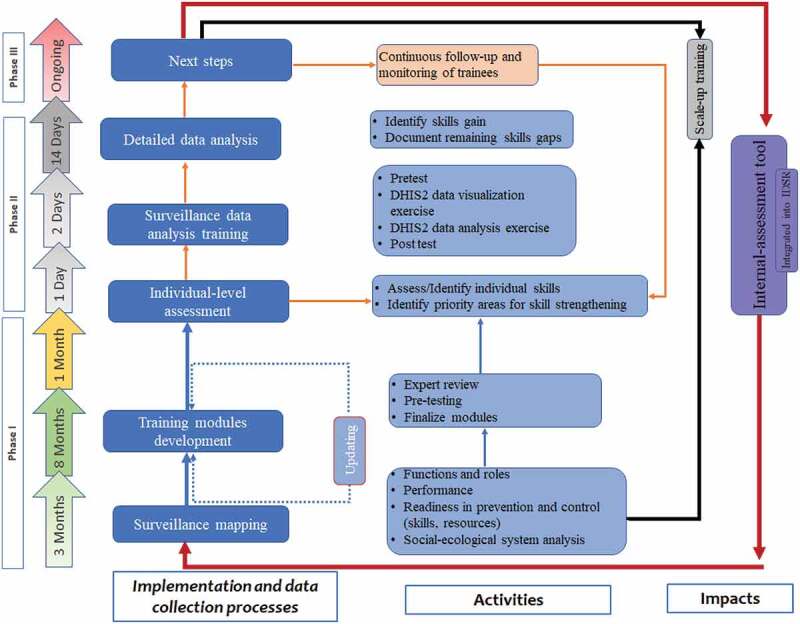
The implementation phases and activities of the capacity-building package.

The entire period for implementing this study was approximately 14 months, from September 2019 to December 2020. The implementation process, the timeline and activities involved are illustrated in Figure 1. Phase I included surveillance system mapping, development of the training modules and preparation of the training workshop and phase II provided detailed information on delivering the training intervention and the analysis done to assess its effect. Phase III was an ongoing process presenting: for the trained teams (follow-up and monitoring), untrained teams (scale up the intervention) and integration of the intervention as a routine IDSR component. The phases and loops were assumed to be done repetitively depending on the identified capacity strengthening needs.

### Mapping of the district surveillance performance

The mapping exercise was done for each district separately. Briefly, information on the performance of IDSR looking at all its components were gathered. This included the structures, human resource availability, roles and responsibility, capacities, readiness of the system to detect, prevent and control diseases with respect to staff and resources, infrastructure, and analytical skills. This exercise involved consultative workshops and discussions with the key members of CHMTs and RHMT.

### Development of the training modules

Based on the gaps identified during the mapping exercise, an intervention package in the form of training modules was developed. The training modules focused on improving the understanding of key statistical concepts used to present epidemiological data, increasing skills in basic data analysis, interpretation, and use of the information for action. The practical exercises during the training were designed to assess the capacity of participants on extraction of set of data on priority diseases from DHIS2, analyse, visualize and interpret, identify alerts or important thresholds and suggests needed response to disease outbreaks in accordance to the Tanzania IDSR Technical Guidelines, 2011 [22]. The modules were shared with disease surveillance experts for critical review and inputs on contents, format, and structure prior to their use. Feedback received was used to refine the materials. The training modules were pretested by conducting a mock training to selected postgraduate students involved in disease surveillance projects. In collaboration with the CHMTs and RHMT members, key participants involved in the management and analysis of surveillance data in Ilala and Kinondoni were identified and invited to attend a training workshop.

### Individual-level assessment

Prior to the development of an intervention package, an individual-level in-depth interview was carried out with the participants to identify skills gaps on surveillance data analysis and use, which included attitudes, motivation, practices and analytical capabilities. A pre-designed semi-structured interview guide was converted to a questionnaire then installed in smartphones with *AfyaData* app [23] to capture the collected data. The questions covered the following: (i) perception on the value of information, the importance of incentives to perform analysis and response, and self-efficacy; (ii) motivation to analyse and interpret data; (iii) time spent on analysis/interpretation; (iv) challenges faced in implementing data analysis/response tasks; and (v) available policies and guidelines related to disease surveillance and data analysis. Using the five‐point Likert scales (‘strongly agree’, ‘agree’, ‘uncertain’, ‘disagree’ and ‘strongly disagree’), the participants were asked on levels of agreement on various aspects of data and systems and how they would grade themselves on different skills. The frequency of conducting data analysis task was assessed using a three-point Likert scales (‘frequent’, ‘rare’ and ‘never’). A descriptive exploratory analysis was done on the collected data to generate a baseline understanding of the participants and to identify areas to prioritize during the training sessions.

### Training and data analysis exercise

A training workshop was preceded by a pre-test that asked questions on knowledge related to disease surveillance, key steps in data management, importance of public health surveillance, and participants understanding on data analysis, epidemic/action thresholds, its importance, measures used in summarization, and ways to present/communicate epidemiological data. The same questions were administered as a post-test after the training intervention.

Two hands-on practical exercises on utilizing data extracted from DHIS2 were embedded during the training, one led by the facilitators and the other by the participants teaming up in their respective districts. Prior to these practical exercises, the participants and facilitators discussed and agreed on one epidemic-prone disease category (referred here as Category A) and one disease of public health importance (Category B), as exemplar tracers. Viral Haemorrhagic fevers (VHF) and pneumonia were selected for Category A and Category B, respectively. Data for these diseases were extracted, analysed, interpreted and the experience of the practical exercise were discussed in plenary sessions.

The surveillance data management components assessed were performance in extraction, visualization, presentation, interpretation, detection of the epidemic thresholds, action taken and suggested future actions. For Category A, deliberate exploration was made as to whether the reported incidence of VHF prompted any action from the districts. The team agreed to assess data covering at least the past 3–5 years. Details of the exercise including expected outcomes based on the IDSR guidelines are given in Box 1 for Category A and Category B diseases.

**Box 1.** Details on setup of practical exercise to strengthen district capacities in surveillance data analysis, interpretation and use¥.


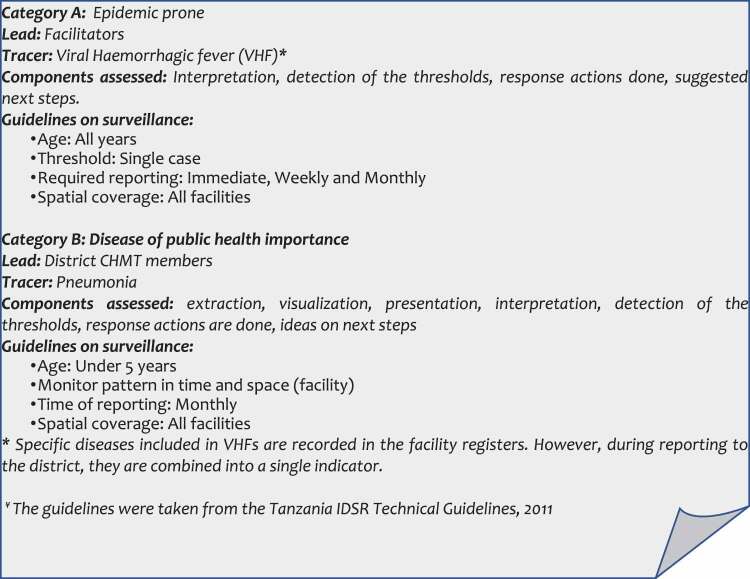

### Data management and analysis

#### Individual-level assessment

**Figure 2.**
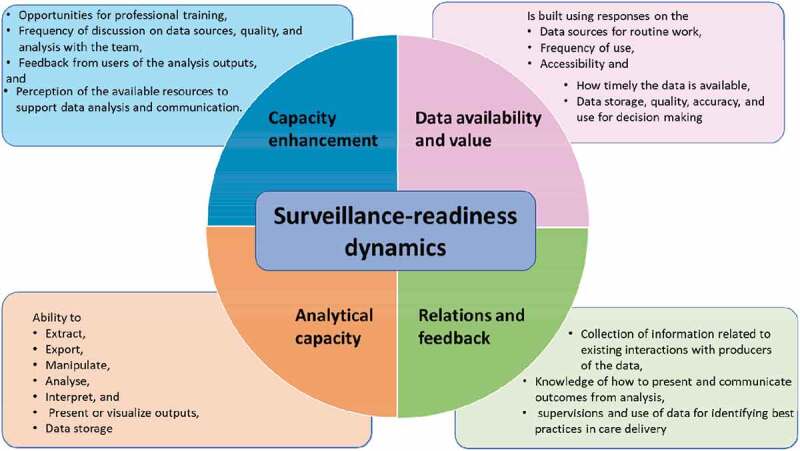
Surveillance readiness dynamics in data availability, analytical capacity, relations and feedback and capacity enhancement.

Data collected from the individual-level assessment was submitted to a server. Quality assessment was conducted by examining different fields of the questionnaires and their completeness. The pre-and post-tests data were analysed by comparing scores at the individual and district levels to assess knowledge and skills gained. The scores from the individual-level assessment, pre- and post-tests were triangulated to derive an understanding on the performance of the surveillance system in the districts and between the two districts. To perform the analysis, the questions from the individual assessment were used to create four surveillance-readiness dynamics: data and information value; analytical capacity and motivation; relations and feedback; and opportunity for enhancing capacity. Components and attributes included in each dynamic are shown in Figure 2.

The questions with more than three Likert scales were collapsed into three categories to create levelling of the distribution of the scoring. To create meaningful total scores that are closely consistent with the participant’s interpretation another decoding was introduced to the scores by making values of agreement/ above average/ Frequent = 1, neutral/ average/rarely/ = 0 and disagreement/ below average/never = −1.

Individual’s average score for each of the surveillance-readiness dynamics was calculated by dividing the score by the maximum value expected for that dynamic based on the number of questions included. For instance, if 10 questions were used to form a dynamic then the maximum expected total score is 10; if an individual scores is 5, the average score will be 50%. The overall scores by district were then calculated and used to assess performance.

#### Pre- and post-training scoring

The questions from the tests were grouped into five surveillance-knowledge categories: general understanding of disease surveillance and its activities; the what’s, whys and how’s (WWHs) of epidemiology and public health surveillance; the WWHs of data analysis; common epidemiological measures (mean, proportions, percentages and event patterns); and uncommon ones (median, rates and ratios).Average scores for each of the categories were calculated and compared between districts. Percentage differences between the mean scores were calculated and used to measure variation between district, i.e. indicating how closer or spread the individual scores of the two districts lie. For the practical exercise on data analysis a detailed assessment comparing expected practices according to the IDSR guidelines on how to respond to the two diseases (Box 1) was done. Best practices, challenges and other important observations were recorded and discussed.

## Results

### Mapping of the surveillance system

Twenty-seven key district officials were involved in the mapping of the surveillance system. Findings from the mapping exercise indicate that the two districts reported to perform well in terms of health facility completeness and timeliness in weekly and monthly surveillance reporting. The challenges in disease surveillance included both intrapersonal and interpersonal factors, lack of guidelines in case management, difficult language used in standard case definitions, inadequate laboratory capacity, lack of appropriate rapid response teams, inadequate knowledge on outbreak investigation and inadequate capacities in data management.

It was reported that the surveillance officers were not skilled enough on data entry, manipulation, and analysis. Participants admitted on lack of feedback on data analysis. They complained about not been able compare current and previous data due to lack of consistency. There was no sharing of information, knowledge, skills and experience between the surveillance and HMIS officers on data analysis at district level. In addition, they reported the existence several health information systems for district to use. These included Health Management Information System, National System for Government Health Officials and the Government of Tanzania Hospital Management Information System. It was proposed by the participants that there should be integration of surveillance system data to HMIS. Inadequate periodic supportive supervision from higher levels was also expressed by the participants.

Several guidelines and reports were available and accessed for review. These included the National Guidelines for Integrated Disease Surveillance and Response 2011, Guidelines on Data Analysis and Use, and Guidelines on Monitoring and Evaluation. However, none of the two districts had Health Information Systems Policy Guidelines. Records on disease surveillance available at the district office included: (i) Weekly summary reports from health facilities; (ii) Monthly summary reports from health facilities; (iii) Weekly summary reports submitted to regional and national levels; and (iv) Monthly summary reports submitted to the regional and national levels. Computers, including desktops and laptops were available and used for report writing, data entry and storage. Ilala district had more computers (33) than Kinondoni (18), though most of them (Ilala) were owned by the staff themselves (15).

### Individual-level assessment

The interview involved 21 participants (females = 10; males = 11). The overall median age of the participants was 44.5 years (IQR = 37, 53). Fourteen (66.7%) had attained university education (Bachelor degree = 7; Master degree = 7) education while others had attained advanced diploma (3), ordinary diploma (3) and secondary school education (1). The overall median number of years of working experience was 5 (IQR = 3, 15). Two participants had less than seven months of working experience. The positions of the participants in their respective districts were the IDSR Focal Persons (3), Maternal and Child Health Officers (3), Health Research Coordinators (2), Laboratory Technologists (2), Health Management Information System Focal Persons (2), Environmental Health Officers (2), Malaria Focal Persons (2), Tuberculosis and Leprosy Coordinators (2), Immunization and Vaccine Development Coordinators (2), and Sanitation and Hygiene Coordinator (1).

The participants from the two districts demonstrated similarity on how they perceived the level of availability and values of the data used for surveillance with an average agreement of about 60% (difference between the district score = 0.46%). In both districts, over half of the participants regarded their analytical capacities and skills as excellent. However, a good proportion of the participants placed themselves in the low capability category. Excellent skills reported included data manipulation, interpretation, and developing of graphs to assess temporal trends. Very few reported to have skills to produce maps. A slight difference (9.26%) was observed for the agreement on the presence of analytical capacity between the two districts with Kinondoni presenting higher average score (61%) than Ilala (52%) (Table 1).

**Table 1.** Performance of surveillance programme dynamics by district.

Dynamics	Ilala			Kinondoni			% Difference between districts’ means
	Average	Minimum	Maximum	Average	Minimum	Maximum	
Data availability and value	59.7%	35.4%	70.8%	60.2%	37.5%	70.8%	0.46%
Analytical capacity	51.9%	12.5%	83.3%	61.1%	33.3%	83.3%	9.26%
Relations and feedback	63.6%	22.7%	90.9%	83.3%	50.0%	100.0%	19.70%
Capacity enhancement	68.4%	30.8%	100.0%	91.5%	61.5%	100.0%	23.08%

The major sources of data for IDSR in the two districts were reported to be routine weekly and monthly morbidity and mortality reports from health care facilities extracted from the HMIS. Among the challenges reported included data incompleteness, long time periods spent to find out the required data for timely decisions, difficulties in the way the data was stored which hinders access. Data were reported to be used for monitoring interventions; decision making; budget preparation; setting targets and goals; ordering of medical supply and drug management; planning, supervision, health promotion and assessing health facility performance. The software/programmes reported to be used for routine data analysis included Microsoft Excel and DHIS2.

Overall, Kinondoni presented higher scores than Ilala in all dynamics. Although almost two-third of the respondents from Ilala and over 80% from Kinondoni reported to frequently interact with data producers/sources (respective health facilities) and other users, conducted joint discussions on data and feedbacks regarding data analysis outputs, a significant difference was observed between the districts (difference of 19.7%) (Figure 2). Agreement on the availability of the opportunities to enhance capacity and skills was reported by two-third of the participants from Ilala (68%) and 91% from Kinondoni translating to a between district difference of 23.1%. Professional training related to enhancement of data management and analysis was reported to be infrequently done. Only a few respondents reported to seeking advice in terms of data management from the higher levels (regional/national).

### Pre- and post-training performance

In general, participants performed better on diseases surveillance questions than on data analysis questions. The overall pre-training score for Ilala was 65.7% while it was 74.0% for Kinondoni. The question with lowest score (average 18% in both districts) was on the usefulness of descriptive images in public health surveillance. As regards to surveillance knowledge categories, Kinondoni indicated a slightly higher scores in almost all the categories than Ilala, with a significant low minimum score observed in the general understanding of surveillance in Ilala (minimum value = 27% vs. 64%) and in the epidemiology and public health concepts (minimum value = 55% *versus* 73%). Interestingly, the overall percentage difference was similar in all categories, indicating a homogeneity in the overall performance of the districts.

The overall post-training average scores were 90.5% for Ilala and 89.1% for Kinondoni. Ilala scored slightly higher values in all categories except the general understanding of surveillance. However, a minimum score of 40% was obtained by one participant in the general understanding of surveillance. The measure of variation, the percentage difference was small in epidemiological concepts, data analysis and common epidemiological measures. However, it seems the training had little impact on the knowledge in common epidemiological measures in both districts. Combining all questions, the overall percentage increase between pre- and post-training indicated a percentage increase of 37.6% for Ilala district and 20.4%. for Kinondoni district indicating a positive impact on participants’ knowledge on surveillance (Table 2).

**Table 2.** Pre- and post-training scores and percentage difference increase by district.

		Pre-test			Post test			Post – Pre	
Category		Ilala	Kinondoni	Diff	Ilala	Kinondoni	Diff	Ilala	Kinondoni
General understanding									
Mean		84.1%	90.3%	6.3%	91.3%	93.1%	1.9%	7.2%	2.8%
Minimum		27.3%	63.6%		40.0%	80.0%			
Maximum		100.0%	100.0%		100.0%	100.0%			
The WWH of epidemiology and public health									
Mean		63.6%	75.0%	11.4%	95.0%	90.0%	5.0%	31.4%	15.0%
Minimum		54.5%	72.7%		90.0%	85.0%			
Maximum		72.7%	77.3%		100.0%	95.0%			
The WWH of data analysis									
Mean		75.8%	87.9%	12.1%	93.3%	91.7%	1.7%	17.6%	3.8%
Minimum		63.6%	72.7%		90.0%	75.0%			
Maximum		86.4%	100.0%		100.0%	100.0%			
Common epidemiological measures									
Mean		41.8%	49.1%	7.3%	87.0%	86.0%	1.0%	45.2%	36.9%
Minimum		22.7%	18.2%		70.0%	80.0%			
Maximum		72.7%	90.9%		100.0%	100.0%			
Uncommon epidemiological measures									
Mean		54.5%	61.4%	6.8%	88.8%	85.0%	3.8%	34.2%	23.6%
Minimum		22.7%	27.3%		70.0%	75.0%			
Maximum		81.8%	81.8%		95.0%	95.0%			

Diff. = Difference; WWH = What, Why and How

### Surveillance data analysis, interpretation and visualization

**Figure 3.**
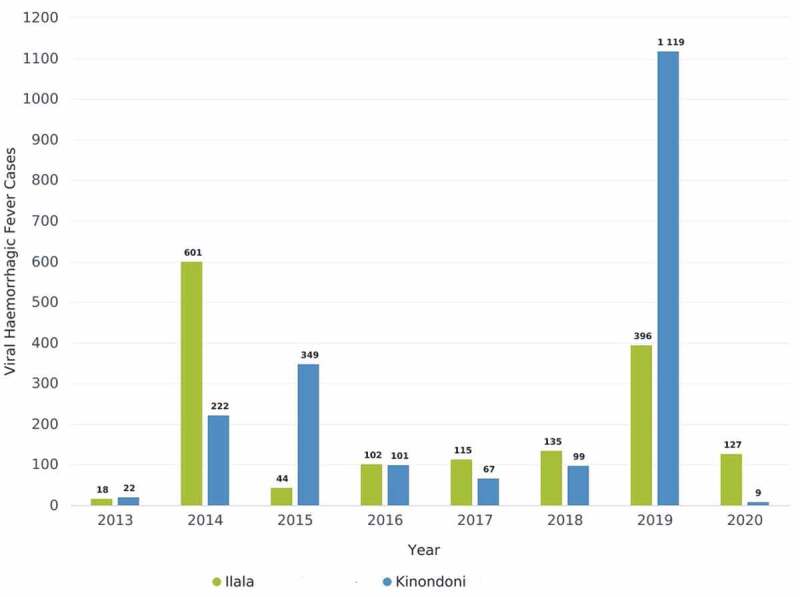
The number of reported cases of VHF in Ilala and Kinondoni districts, 2013–2020.

**Figure 4.**
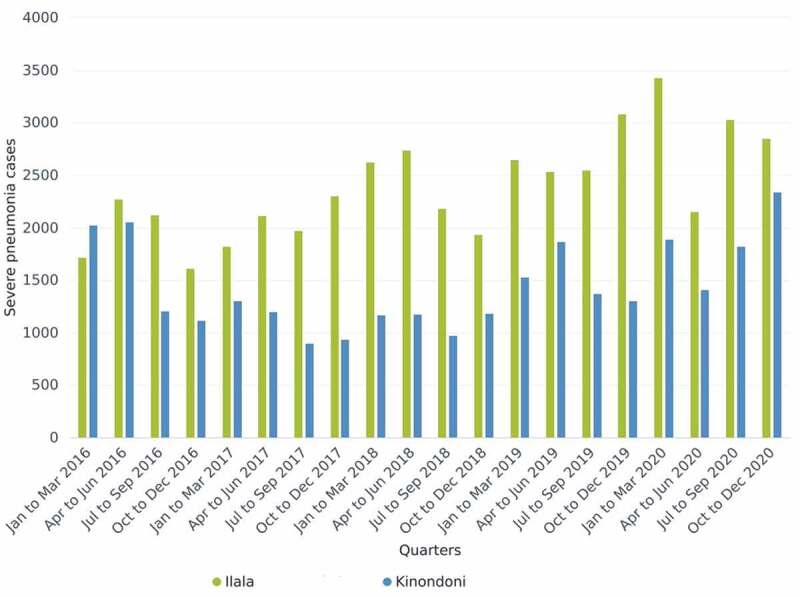
Quarterly number of cases of severe pneumonia in Ilala and Kinondoni districts, 2016–2020.

The practical exercise was conducted for the two categories, epidemic prone diseases using VHF (category A) and disease of public health importance using pneumonia (category B). Data for VHF (Figure 3) were extracted from DHIS2 by the facilitators while those for pneumonia (Figure 4) were extracted by the district teams. Graphs were developed and presented during plenary discussion. Two of the facilitators documented all reactions from the participants in relation to the presented data.

The VHF data triggered a discussion on whether or not these were the correct data submitted from the health facilities, and if so, what exactly were the actions taken by the district authorities.

During discussion, it was observed that though the district team noted that the VHF cases were reported from healthcare facilities during the whole period under review (2013–2020), no analyses were ever done. Moreover, despite the reported VHF cases for over 5 years and with increasing trend over time, and the fact that a single case of VHF requires notification to the higher level and actions to be taken, the district teams acknowledged that none of the two actions (including outbreak investigation and response) were taken as required by the surveillance guidelines (Table 3).

**Table 3.** Assessment of VHF data analysis, expected actions and expected actions by district.

Variable	Expected action	Ilala	Kinondoni
Attribute			
Threshold for action	Single case	Threshold met	Threshold met
Age category	All years	Analysis by age not determined	Analysis by age not determined
Spatial coverage	All facilities must be included	Affected areas/facilities not determined	Affected areas/facilities not determined
Required reporting	Immediate, Weekly and Monthly	Data available in the system	Data available in the system
Required action	Immediately within 24 hours	None	None
Assessment criterion			
Interpretation	Outbreak if threshold met	Outbreak occurred in all years	Outbreak occurred in all years
Detection of the thresholds	Guided by threshold	Not done	Not done
Response	Respond if a case reported	Not done	Not done
Suggested next steps	Continuous monitoring	Improve alert system	Improve alert system

Data on severe pneumonia cases from 2016 t0 2020 were analysed the quarterly trend presented to the participants for interpretation exercise and discussion. Although one of the requirements of the IDSR Guidelines is to analyse disease incidence trend in space and time, the district teams admitted that this is not routinely done, and it was their first time to notice the pattern of pneumonia in the districts. Through discussion, it could not be established as to the reasons for the pattern of pneumonia in the two districts. These data triggered a discussion on the number of cases reported, long-term changes in disease event occurrence, reasons for changes in disease occurrence, and comparison on the number of cases reported for different slots of time in quarters, seasons or years. The results of this exercise and discussion allowed users to add knowledge and skills on data extraction, visualization, presentation, interpretation, detection of the thresholds, response actions (Table 4).

**Table 4.** Assessment of severe pneumonia data analysis and the expected actions by district.

Variable	Expected action	Ilala	Kinondoni
Attribute			
Threshold for action	Number of cases for the period clearly exceeds cases of previous year/season	Threshold met	Threshold met
Age category	Under 5 years	Analysis by age not determined	Analysis by age not determined
Spatial coverage	All facilities must be included	All facilities were included	All facilities were included
Required reporting	Monthly, Quarterly	Data available in the system	Data available in the system
Required action	Appropriate treatment at health facility, appropriate and rapid referral for hospitalization	Follow up check was not done	Follow up check was not done
Assessment criterion			
Interpretation	Outbreak if threshold met	Outbreak occurred in some quarter	Outbreak occurred in some quarter
Detection of the thresholds	Guided by threshold	Not done	Not done
Response	Respond if an outbreak reported	Not done	Not done
Suggested next steps	Continuous monitoring	Improve alert system	Improve alert system

## Discussion

Generally, in the two districts, the overall performance of the disease surveillance system in terms of detection, monitoring and response to disease outbreaks wa not satisfactory. There were inadequate skills and capacity in data management including data analysis, interpretation and use, despite the availability of the guidelines on surveillance data analysis and use in both districts. Previous studies that in low- and middle-income countries, including Tanzania, reported a limited or no evidence of routine data analysis at the sub-national levels mainly due to lack of clear guidelines on how and when to analyse data [7,24–28].

The absence of data analysis, interpretation and utilization at the sub-national level was observed in the present study is in line with findings of studies in Ethiopia, Malawi and Nigeria [29–31]. Skill gap in data management system, weak supervision and feedback system, low or no legal enforcement to the surveillance activities, lack of incentives, lack of continued capacity strengthening, and lack of sense of ownership have been reported as factors affecting analysis and use of surveillance data [7,30,31].

The trained district personnel are key in the performance of the national IDSR, as district is the crucial link between data source (healthcare facility) and decision/ policy makers (ministry level). Studies elsewhere have also reported that despite improvements in technological solutions and introduction of DHIS2, the actual use of data remains limited, especially at lower levels such as the facility and district in many countries [17,32]. In another recent analysis of the health information systems at the district level major gaps in data analysis, interpretation and use have been identified [7].

The findings from individual level assessment indicate that over a quarter of the participants from the two districts reported some difficulties in finding out the required data to make timely decisions. Although a good number of the participants regarded their technical skills in data management as excellent, they claimed to be less motivated on issues related to data management cycle. Despite all these, data in the two districts was described to be used for planning. The use of data in the identification of emerging epidemics was only mentioned in one district. Studies in Tanzania and elsewhere have reported that inadequate resources, lack of analytical and data use skills, refresher courses and review meetings, pressure of work, lack of incentives for data use are the most important systems-level management challenges in HMIS [30,33].

As regards to monitoring of disease occurrence in the district, only a few of the members were aware that their respective facilities reported notifiable epidemic diseases, including VHF during the past five years. Although all participants agreed that it is important to regularly examine the data submitted to the district level for any unusual event, none of them was prompted to take action in response to the VHF cases reported. The observations in the two districts emphasize the need for alert thresholds be applied to notifiable disease surveillance data to provide guidance for triggering further actions. The thresholds should be able provide an alert when the number of cases of a specific disease exceeds a pre-established threshold. However, this can only be done, if those responsible for routine analysis and interpretation of surveillance data do their job. During discussion, it was noted that the two districts actually experienced dengue epidemics during the reporting period, but since the variable in the DHIS2 system was VHF, the specific disease was not recorded. Tanzania, and particularly the City of Dar es Salaam has experienced dengue outbreaks during 2013–2015 and 2019 [34,35]. It is therefore important that the disease surveillance guidelines should emphasize the need for reporting of specific disease rather than groups of diseases.

According to the National IDSR Guidelines, one single case of VHF is enough to initiate an investigation [22], yet none of the district did this. Alert thresholds must take into consideration the local epidemiology and will vary for different diseases and in different settings, depending on disease severity and epidemic potential. It is important that an electronic diseases early warning system is incorporated in the DHIS2 to strengthen routine early warning detection of epidemic-prone diseases, thus provide an alert and facilitate rapid response [36,37]. Such early warning systems are useful tools for early detection and prompt response to outbreaks.

Fundamental challenges facing analysis and interpretation of surveillance data have been identified to include: understanding the purpose and context of the specific surveillance system; identifying a baseline rate of observations and to recognize deviations from that baseline; interpreting the meanings conveyed by these observations and to recognize the significance of these interpretations; properly discerning the degree of certainty that the available data can support regarding that interpretation; and timely communicating the observations with clarity to the desired actors that enables meaningful actions to be taken in response to the interpreted data [38]. These findings clearly indicate that, it is not enough to collect and report morbidity and mortality data from the facilities. Such data must be analysed in terms of time, place and person at each surveillance level [31]. It should be realized that if the district doesn’t analyse and use the data, the utility of the surveillance system becomes minimal, which makes the system too weak to pick outbreaks early enough to guide prompt response [31]. The possible reasons for lack of data analysis might include skill gap in data management system, weak supervision and feedback system, low or no legal enforcement to the surveillance activities, lack of incentives and continuing capacity building programmes, as well as lack of sense of ownership [7]. A functioning surveillance system is expected to enable decision makers at all levels of the health system to monitor progress, identify unusual events, make evidence-based decision on programmes and allocate resources [39,40].

The design and process of the training programme implementation provided an invaluable contribution to the responsibilities of the district teams. Firstly, the data analysis training brought together health workers of various disciplines and disease control programmes. This helped health workers who are primarily not involved in surveillance activities appreciate their indirect yet critical contribution to the surveillance programme and helped foster teamwork. Bringing together such a team aimed to ensure that competent health teams that can readily be mobilised and deployed for rapid outbreak investigation and response are available within the districts. Similar approaches have been reported to contribute to improvements in data use in Zanzibar and Cote d’Ivoire [41,42]. The evaluation of the impact of the training indicate that there was an improvement in knowledge of the epidemiology and disease surveillance parameters among the trainees. Generally, this post-evaluation performance clearly indicate that the training had a positive impact on participants’ knowledge on surveillance and data analysis for both districts. Although training in this study was evidently found to contribute substantially in motivating district team members in realizing the value of surveillance data, it should be noted that training alone is insufficient to engage and build capacity for district health workers. Stakeholder meetings, data reviews, and mentored use of data in decision making are equally important to engage health workers and managers and demonstrate the value of data [42].

One strength of this study is the involvement of the district health personnel in the data analysis exercises, suggesting that the findings have relevance throughout the health system in Tanzania. A potential limitation of this study was the small key informant sample. However, this part of the study was designed to prepare the background for the in-depth qualitative assessment rather than to yield statistically representative results. Moreover, since this study involved urban districts only, the findings cannot be used to generalize the situation in the rural district.

## Conclusions

The findings that epidemic-prone diseases were reported by the facilities and yet the districts did not take measures to analyse and investigate, demonstrate the need to strengthen the capacity of the district in the use of alert systems to identify the occurrence of notifiable diseases. The experiences from the two districts highlight the need to take conscious action to address knowledge and skill gaps of the human resource in order to improve performance in disease surveillance. Districts should introduce disease surveillance as an agenda in their routine meetings to examine reporting of unexpected disease and unusual trends observed over time. Like many complex health system interventions, success in the improvement of data management and use in decision-making should take a whole-stakeholder involvement approaches that involve the frontline health workers, district managers and policy-makers to realise the need and value of data. For surveillance to have an impact on health system performance, and hence improvement of the population health, it is important to address the district health workers culture and norms around data that will make the difference in the way they value data. Periodic refresher trainings are important and likely to make the surveillance system more user-friendly.

## Data Availability

The data of this study are presented in the main manuscript. Any additional supporting files are available from the corresponding author on reasonable request.

## Paper context

Disease Surveillance data analysis and use for decision making is inadequate in Tanzania. This is due to inadequate capacities at levels. The performance of the district surveillance system was assessed; and based on identified gaps, an intervention package was developed. Following training, the overall surveillance knowledge increased by 20.4 to 37.6%. The training significantly improved skills of the participants; demonstrating the need to strengthen the capacity of the district in data analysis and use.
